# Estimation of HIV-1 incidence among five focal populations in Dehong, Yunnan: a hard hit area along a major drug trafficking route

**DOI:** 10.1186/1471-2458-10-180

**Published:** 2010-04-07

**Authors:** Song Duan, Sheng Shen, Marc Bulterys, Yujiang Jia, Yuecheng Yang, Lifeng Xiang, Fei Tian, Lin Lu, Yao Xiao, Minjie Wang, Manhong Jia, Huazhou Jiang, Sten H Vermund, Yan Jiang

**Affiliations:** 1 Department of HIV/AIDS Control and Prevention, Dehong Dai and Jingpo Nationality Autonomous Prefecture Center for Disease Control and Prevention, Dehong, Yunnan, PR China; 2 National AIDS Reference Laboratory, National Center for AIDS/STD Control and Prevention, Chinese Center for Disease Control and Prevention, Beijing, PR China; 3 Global AIDS Program-China, US Centers for Disease Control and Prevention; 4 Amos Christie Chair of Global Health, Institute for Global Health, Vanderbilt School of Medicine, Nashville, USA; 5 Department of HIV/AIDS Control and Prevention, Yunnan Provincial Center for Disease Control and Prevention, Kunming, Yunnan, PR China

## Abstract

**Background:**

Since 1989 when the first 146 HIV positives in China were identified, Dehong Prefecture had been one of the areas hardest-hit by HIV in China. The local and national governments have put substantial financial resources into tackling the HIV epidemic in Dehong from 2004. The objective of this study was to track dynamic changes in HIV-1 prevalence and incidence among five focal populations in Dehong and to assess the impact of HIV prevention and control efforts.

**Methods:**

Consecutive cross-sectional surveys conducted in five focal populations between 2004 and 2008. Specimens seropositive for HIV were tested with the BED IgG capture enzyme immunoassay to identify recent seroconversions (median, 155 days) using normalized optical density of 0.8 and adjustments.

**Results:**

From 2004 to 2008, estimated annual HIV incidence among injecting drug users (IDUs) decreased significantly [from 15.0% (95% CI = 11.4%-18.5%) in 2004 to 4.3% (95% CI = 2.4%-6.2%) in 2008; trend test P < 0.0001]. The incidence among other focal populations, such as HIV discordant couples (varying from 5.5% to 4.7%), female sex workers (varying from 1.4% to 1.3%), pregnant women (0.1%), and pre-marital couples (0.2 to 0.1%) remained stable. Overall, the proportion of recent HIV-1 infections was higher among females than males (P < 0.0001).

**Conclusions:**

The HIV epidemic in Dehong continued to expand during a five-year period but at a slowing rate among IDUs, and HIV incidence remains high among IDUs and discordant couples. Intensive prevention measures should target sub-groups at highest risk to further slow the epidemic and control the migration of HIV to other areas of China, and multivariate analysis is needed to explore which measures are more effective for different populations.

## Background

Dehong Prefecture lies on the China-Myanmar border in Yunnan Province, which is one of the areas hardest-hit by HIV in China [1]. Since 1989 when the first 146 HIV-infected injection drug users (IDUs) were identified in Dehong, this area has reported a cumulative total of 14,903 HIV/AIDS cases through 2008, representing 6.2% of China's reported HIV/AIDS cases though it has only 0.08% of China's population.

According to sentinel surveillance data, IDUs represent the largest single group of HIV-infected persons in Dehong [1]. From IDUs, the epidemic has spread to other groups, particularly female sex workers (FSWs). Sexual transmission is now the most common mode of HIV transmission in Yunnan Province [2]. In order to limit transmission of HIV to a minimum, a number of targeted intervention services including needle exchange programs, methadone maintenance therapy, condom distribution, voluntary counseling and testing, antiretroviral therapy, prevention of mother-to-child transmission and educational information about drug use and HIV have been introduced [3]. Since 2004, the local and national governments have put substantial financial resources into tackling the HIV epidemic in Dehong. In order to assess the impact of HIV prevention and control efforts, we examined the trend in HIV incidence among five focal populations: IDUs, discordant couples, FSWs, pregnant women, and couples going for pre-marital counseling and testing. These five populations represent the main targeted incidence surveillance populations in Dehong during this time period.

The incidence of HIV has recently been estimated using laboratory-based assays that distinguish between recent and long-term HIV infection. This method avoids some of the biases inherent in testing participants repeatedly in a prospective cohort where the actual follow-up process can influence behaviors. Specimens are collected from serial cross-sectional surveillance surveys [4]. In the present study, we used the BED capture enzyme immunoassay (BED-CEIA) for the detection of recent HIV infection and the calculation of incidence estimates [5-7]. The assay (using suitable adjustments described in the methods) has previously been validated in study populations in China, in which the difference between HIV incidence estimated by BED and prospective cohort studies was similar [8,9]. BED has proven sensitive for the detection of HIV recent infection but are vulnerable to misclassifying established infections as recent [10], though test efficiency can be improved with adjustments for false positives due to low CD4+ cell count, antiretroviral use, and advanced clinical disease [11-13]. This study explored the HIV incidence trend in Dehong to assess the impact of HIV preventive efforts and to guide future prevention and control efforts in China.

## Methods

### Study design and target population

Consecutive cross-sectional surveys were conducted each year between 2004 and 2008 among IDUs, discordant couples, FSWs, and pregnant women and compared with sentinel surveillance site trend data over the same time span. Between 2006 and 2008, couples presenting for pre-marital counseling and testing were also included to represent young adults in the general population.

#### IDUs

Serial cross-sectional surveys were conducted in all five detoxification centers in Dehong (there is one detoxification center in each of five counties: namely Lianghe, Luxi, Longchuan, Ruili and Yinjiang). IDUs at the detoxification centers are estimated to represent about 25% of all drug users in Dehong Prefecture.

#### Discordant couples

The partners of HIV positive individuals were recruited into the discordant couple group if they tested HIV seronegative.

#### Female sex workers

Data from FSWs were collected annually from five cities and counties in Dehong at the FSWs' workplace. There were an estimated 160 fixed and 10 flowing FSW workplaces in Dehong by the end of 2008. More than 85% of FSWs in Dehong were believed to work at fixed sites.

#### Pregnant women

HIV counseling and testing was implemented for all pregnant women at each of the 3 levels of the health care system (county MCH hospital, township hospitals, and village health centers) in Dehong. In 2008, an estimated 95% of pregnant women accepted HIV testing in antenatal clinics.

#### Couples in pre-marital testing

From 2006, routine 'opt-out' HIV testing was added to standard pre-marital health check-ups in hospitals in five counties, and the acceptance rate for HIV testing was more than 95%.

### Data collection

The questionnaires were designed and administered by staff from the Prefecture Center for Disease Control (CDC). Oral informed consent was obtained for the questionnaire and for the blood collection from all participants, with the interviewer certifying oral consent by signing the form. The data were collected as part of routine HIV surveillance activities, including information on behavioral, social and demographic indicators and ART use. Clients were informed as to the purpose of the blood test and conventional HIV antibody test results were provided to all subjects. While a large proportion of approached subjects agreed to testing, we do not have precise data on the number of refusals. All HIV-positive patients were referred for free HIV care and treatment according to the China "Four Frees and One Care" policy [14]. The study was approved by the Institutional Review Board of the Yunnan CDC.

### Laboratory methods

Two enzyme-linked immunosorbent assays (Anti-Human Immunodeficiency Virus 1+2 Antibody ELISA Kit, Beijing BGI-GBI Biotech Co., Ltd., Beijing, China) and a Western blot (Genelabs Diagnostics Pte. Ltd., Singapore) confirmatory test were performed to determine HIV status. HIV-positive patients were also checked for CD4+ T cell count. HIV-positive patients for whom the CD4+ T cell count was ≤200 and who self-identified as on antiretroviral treatment (ART) were excluded from further HIV incidence testing. The known long-term HIV infection and AIDS cases were also not included. All other HIV positive specimens were tested for recent infection using BED-CEIA, as described elsewhere [15]. Samples positive on BED assay testing were categorized as recent infection. Results from BED tests were not provided to participants because the assay has only been validated for population-level testing [11,16].

### Data Analysis

Survey and serological data were independently double-entered into EpiInfo™ software (version 3.4.3-CDC-Atlanta, GA, USA). Participants' data were cross-referenced with China's Information System for HIV/AIDS Prevention and Control national HIV surveillance database. Participants were determined not to have new infections if their name, birthday, and address matched a person already entered in the national HIV system. Data were evaluated for congruency using EpiData software (EpiData 3.0 for Windows™, The EpiData Association Odense, Denmark). Statistical Program for Social Sciences (SPSS, version 15.0) software was used for all statistical analyses. The BED-estimated HIV incidences were calculated by a Microsoft Excel-based program for automated results interpretation prepared for the study according to Calypte's instructions. An ODn of 0.8 corresponds to mean seroconversion duration of 155 days and the samples with an ODn of <0.8 are considered to be from individuals with recent infection. Adjustment of the incidence formula was performed according to Hargrove et al. [13]. Specifically, adjustment factors γ and ε (γ = 0.9348, refers to specificity of the BED test over the period >2 times the window period, ε = 0.0652, refers to the rate of recent false-positives in those with >2 times the window period, γ + ε = 1) were previously found to be suitable for HIV incidence estimation of diverse populations in China [8].

## Results

**Figure 1:**
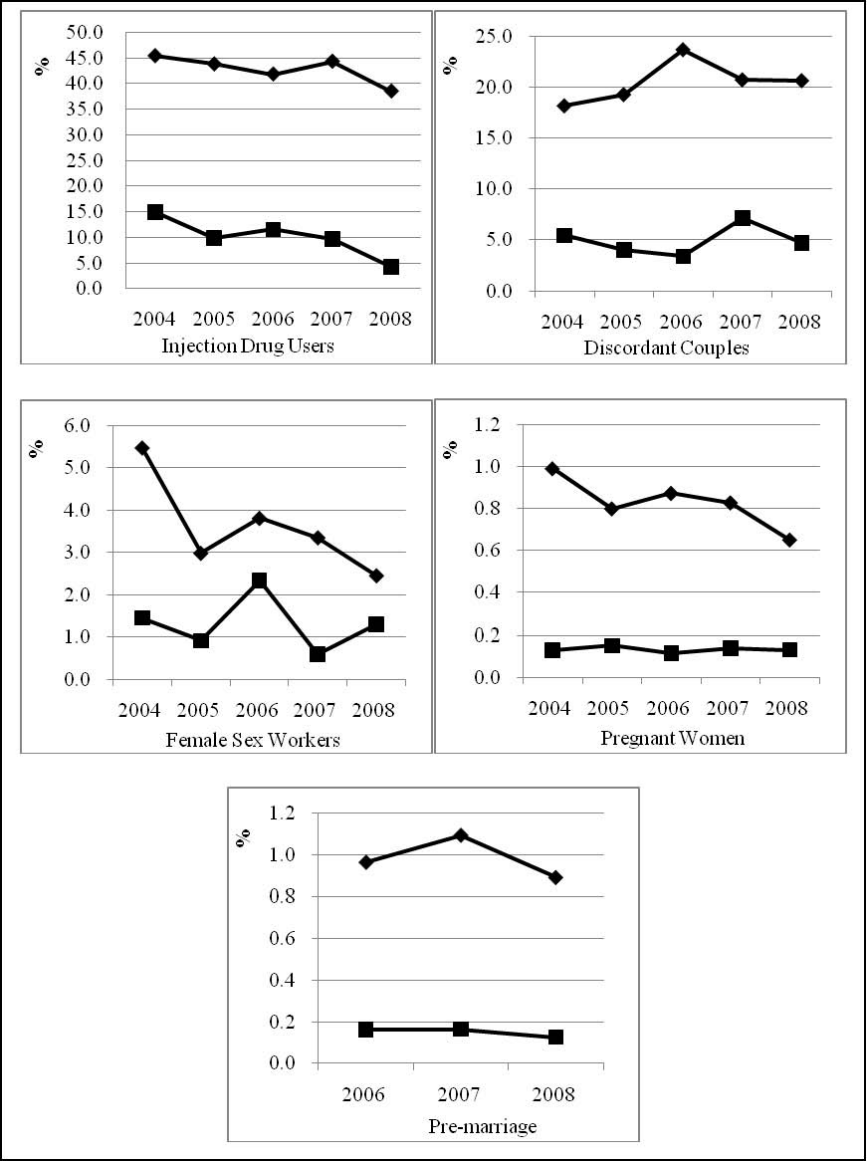
**HIV prevalence and incidence among five populations in Dehong Prefecture, Yunnan Province from 2004 to 2008**. Note: Black rhomb indicates HIV-1 prevalence; black square indicates HIV-1 incidence.

The total number of people tested for HIV in each of the five populations increased from 8084 in 2004 to 44,129 in 2008 (Additional file 1). The total number of pregnant women tested increased from 4,944 in 2004 to 20,958 in 2006 and remained stable thereafter. Pre-marital couples were only offered HIV testing from 2006-2008. Prevalence and incidence estimates were obtained annually for each of the five focal populations (Figure 1).

### IDUs

**Figure 2:**
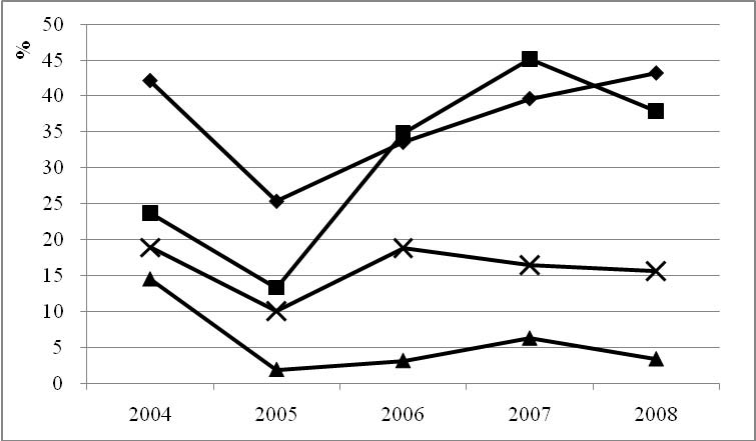
**Prevalence of sexually transmitted diseases, self-reported commercial sex and risk behaviors among injection drug users in Dehong Prefecture, Yunnan Province from 2004 to 2008**. Note: From 2004 to 2008, condom use increased significantly (P < 0.0001), while STD decreased (P = 0.02). Prevalence rates for needle sharing and commercial sex remained relatively stable (P = 0.28 and 0.99). Black rhomb indicates needle sharing; black square indicates condom use; black triangle indicates sexually transmitted diseases; black cross indicates self-reported commercial sex.

The estimated HIV prevalence was 45.4%, 43.8%, 41.8%, 44.3% and 38.4% and the estimated HIV incidence was 15.0%, 9.9%, 11.5%, 9.7% and 4.3% from 2004 to 2008, respectively (trend test, P < 0.0001). HIV prevalence has decreased from 2004 to 2008 (trend test, P < 0.0001); however, it is still high in 2008. Changes in risk behavior among IDUs in Dehong from 2004 to 2008 are displayed in Figure 2. Although needle-sharing and commercial sex were still common, IDUs were more likely to use condoms in the most recent years.

### Discordant Couples

From 2004 to 2008, the HIV prevalence among the sexual partners of HIV-positive individuals was 18.2%, 19.3%, 23.7%, 20.2% and 21.7%, while the estimated incidence was 5.5%, 4.0%, 3.4%, 7.2% and 4.7%, respectively. Neither prevalence nor incidence rates differed significantly over time. Both remained stable at a relatively high level from 2004 and 2008 (trend test, P = 0.23 and 0.75, respectively).

### FSWs

The HIV prevalence among FSW showed a substantial decrease from 5.5% in 2004 to 2.5% in 2008 (P < 0.001), while the estimated incidence was 1.4%, 0.9%, 2.3%, 0.6% and 1.3%, and stayed at a relatively high level throughout the study period (P = 0.45).

### Pregnant Women

The cumulative number of HIV positive pregnant women during the five-year period was 716. HIV prevalence among pregnant women was 1.0%, 0.8%, 0.9%, 0.8% and 0.7%, respectively, while estimated HIV incidence remained stable throughout the five-year period at 0.1%.

### Pre-marital Couples

Between 2006 and 2008, the HIV prevalence for pre-marital couples was 0.96%, 1.1% and 0.89%, while the incidence was 0.16%, 0.16% and 0.13% among pre-marital couples, respectively. Both prevalence and incidence remained stable over time (P = 0.31 and 0.36). The incidence and prevalence among pre-marital couples appeared somewhat higher than among pregnant women but this difference was not statistically significant.

### The proportion of recent HIV-1 infections among all HIV-1 positive participants

(See Additional file 2) Among all HIV-1 positive participants in all groups, the proportion of recent HIV-1 infections among females was higher than that among males (from 12.32% in 2004 to 7% in 2008 for male, and from 15.53% in 2004 to 15% in 2008 for female). As expected for the largest ethnic group, the proportion of recent HIV-1 infections was higher among Han Chinese in 2004 compared to all ethnic minorities, but decreased significantly over time through 2008 (trend test, P = 0.037). Dai and Jingpo are the main minority ethnic groups living in Dehong, and approximately 10% of HIV-positives were recent HIV-1 infections, with no trend over time. Most HIV-1 positive participants were peasants and there was no change over time in the proportion of recent HIV infections among this occupational group. In 2004, a comparatively large proportion (31%) of participants with non-farming occupations had recent HIV-1 infections, though this proportion decreased over time (trend test, p = 0.001).

## Discussion

To our knowledge, this is the largest application of the BED-CEIA to estimate HIV-1 incidence and the first application among discordant and pre-marital couples in China. Simulation of the HIV epidemic among IDUs in Dehong from 2004 to 2008 showed that the epidemic continues to expand but that the rate has been slowing in the most recent years. Our incidence estimates suggest that lower incidence may explain the slowing of the growth of the epidemic among IDUs. The reasons why HIV prevalence among IDUs remained stable while the incidence decreased over the five-year period (but still remained relatively high) may include several factors: 1) A high death rate among HIV-infected IDUs; 2) High mobility among IDUs in Dehong and neighboring areas with lower HIV prevalence; 3) The impact of local government policies on strengthening the use of force to combat drug abuse; 4) Temporal changes in the number of newly developed IDUs and drug addiction levels in different age groups. Finally, misclassification of transmission route may also be a possible explanation.

The epidemic of HIV/AIDS in China consists of at least eight sub-epidemics [17] along with the spread of other STDs, such as syphilis [18]. China's HIV epidemic remains low prevalence overall [19] with high infection among specific sub-populations and in certain geographic areas such as Dehong Prefecture near the border with Myanmar [20]. Until recently, IDU was the leading route of HIV transmission in China and HIV continues to spread rapidly among IDUs in Dehong Prefecture and other major drug trafficking corridors in China [1-3]. However, the HIV transmission mode has been changing gradually from IDU to sexual transmission. Our study demonstrates that HIV incidence among IDUs in Dehong is decreasing. However, incidence among the other four focal populations, all representing sexual transmission of HIV, has remained stable. In our study, sexual transmission of HIV appears concentrated in several high-risk populations (especially discordant couples and FSWs) and has not diffused substantially into the general population.

In a previous study, we showed that IDUs had the highest HIV incidence rate in Yunnan Province, varying between 2.2% and 8.0% from 2004 to 2008, and HIV-1 genetic diversity had become increasingly complex [2]. HIV incidence among IDUs in Dehong was also higher than in other regions of China (except Xinjiang Province in the Northwest) [8]. We speculate that the decrease in incidence among IDUs may be partially explained by the changes in risk behaviors, notably uptake in needle exchange and a reduction in needle sharing [21]. While there has been a notable increase in access to antiretroviral therapy among IDUs and some likely reduction in AIDS- and drug-related deaths, more extensive modeling would be needed to estimate whether or not these have also contributed to lower prevalence and incidence estimates. After IDUs, negative persons in HIV-discordant relationships had the next highest risk of transmission, with incidence estimated by BED varying from 3.4% to 7.2% and is very similar to that in a cohort study of stable sex partners in Africa [22]. Because of the high mobility of FSWs and the fact that many are only in the business for a relatively short period of time, the incidence and prevalence estimates in this group are relatively close.

Since 2004, China Comprehensive Response Project Areas were founded in Dehong, and a series of HIV control and prevention efforts have been initiated, including MMT, needle and syringe exchange, prevention of mother-to-child transmission, and distribution of free condoms as part of a 100% condom policy for sex workers and their clients. Although coverage of intervention programs has rapidly expanded in Dehong, the HIV epidemic was likely influenced differently by these various intervention efforts and we also found that the reported needle sharing by IDUs did not markedly decrease over the period of this study. Major challenges to harm reduction include the high relapse rate for MMT and the limited ability to reach the majority of heroin users due to various barriers [21]. Therefore, further multivariate analysis is needed to explore which measures may be most effective for different populations. Our data emphasize the importance of accelerating HIV prevention programs like needle exchange and MMT to reduce needle sharing among IDUs and condom distribution and promotion to reduce unprotected sex among discordant couples and among FSWs and their clients.

There are a number of different laboratory methods to estimate HIV incidence [23,24] and the use of these methods is still evolving, especially in populations with HIV-1 non-B subtypes. It is likely that BED-CEIA will have its utility as a sensitive screening test, with avidity used to exclude false positives, and modeling to further estimate the remaining pool of false positives. The utility of the BED-CEIA assay has been demonstrated in a number of studies, in particular for elucidating trends in incidence [11,25,26]. Regional data from sub-Saharan Africa on the use of BED have indicated that it overestimates the HIV incidence [27] and further adaptations have been made to enhance accuracy, especially in populations infected with non-B subtypes [13,28]. In the current study, the trend in incidence among IDUs was very similar to that of a prospective cohort study conducted in the same area [29]. The prevalence and incidence among pregnant women and pre-marital couples were very similar, with slightly higher rates among pre-marital couples. These findings indicate that the HIV incidence calculated from the BED-CEIA assay is credible. This has previously been shown also in another study from China [8]. We excluded patients with CD4+ cell count ≤200 and those on ART, as recommended [13]. This could lead to a potential bias of the estimates because relatively less vigorous exclusion was applied in the earlier years than in late years during the period from 2004 to 2008. IDUs recruited from detoxification centers could be an easier target for harm reduction programs than IDUs in the community, which are generally harder to reach; therefore, the trends of HIV incidence among detoxification users may not reflect accurate trends among non-detoxification users. Despite current limitations, methods that allow cross-sectional HIV incidence estimation are an important advance, and these methods will be further refined as more experience is gained in different settings. While absolute HIV incidence estimates may be imperfect, they may still be highly useful to determine trends over time [26,30,31].

## Conclusions

A declining trend in HIV incidence among IDUs in Dehong is encouraging, but with significant numbers of individuals already infected and in need of treatment, further roll-out of HIV care and treatment is needed [32,33]. Prevalence and incidence of HIV infection have stabilized at an unacceptably high level in Dehong Prefecture, and special efforts are needed to reduce sexual HIV transmission and to further increase the coverage of MMT clinics and clean needle-syringe programs [34]. Further efforts are needed to strengthen monitoring and evaluation of the epidemiological situation, to improve the depth, breadth and innovation of intervention methods, to establish supportive policy and legislation, and to strengthen international collaboration and information exchange.

## Competing interests

The authors declare that they have no competing interests.

## Authors' contributions

The first two authors contributed equally to this paper. YJ and SHV initiated the research, DS, YY, LX, LL and MJ conducted the data collection, DS, SS and YX performed the data analysis, SS, MB, YJ and HJ drafted the manuscript, SS, MW and FT participated in the BED test. All authors edited and approved the final manuscript.

## Pre-publication history

The pre-publication history for this paper can be accessed here:

http://www.biomedcentral.com/1471-2458/10/180/prepub

## Supplementary Material

Additional file 1 **HIV prevalence and incidence among five populations in Dehong Prefecture, 2004 to 2008**. From 2004 to 2008, the HIV prevalence among IDU and FSW showed a decrease. And the estimated annual HIV incidence among IDU decreased significantly, while remained stable among other focal populations. Click here for file

Additional file 2 **The proportion of the number of participants with recent HIV-1 infection among HIV-infected participants in Dehong Prefecture, Yunnan Province from 2004 to 2008**. Among all HIV-1 positive participants in all groups, the proportion of recent HIV-1 infections among females was higher than that among males. The proportion of recent HIV-1 infections was higher among Han Chinese in 2004 compared to all ethnic minorities, but decreased significantly over time through 2008. Most HIV-1 positive participants were peasants and there was no change over time in the proportion of recent HIV infections. Click here for file
