# Supplementary material for: Estimation of HIV-1 incidence among five focal populations in Dehong, Yunnan: a hard hit area along a major drug trafficking route
Source: BMC Public Health. 2010 Apr 7;10:180. doi: 10.1186/1471-2458-10-180 (PMC2858119; doi:10.1186/1471-2458-10-180)
Supplement: Additional file 1 — HIV prevalence and incidence among five populations in Dehong Prefecture, 2004 to 2008. From 2004 to 2008, the HIV prevalence among IDU and FSW showed a decrease. And the estimated annual HIV incidence among IDU decreased significantly, while remained stable among other focal populations. [file 1471-2458-10-180-S1.DOC]

| **HIV prevalence and incidence among five populations in Dehong Prefecture, 2004 to 2008** | 95% CI of Incidence | 11.4-18.5 | 6.4-13.3 | 7.8-15.3 | 5.0-14.5 | 2.4-6.2 | 2.9-8.0 | 1.2-6.8 | 1.2-5.6 | 3.8-10.6 | 2.8-6.7 | 0.6-2.3 | 0.0-1.8 | 1.2-3.5 | 0.0-1.2 | 0.4-2.2 | 0.0-0.2 | 0.1-0.2 | 0.1-0.2 | 0.1-0.2 | 0.1-0.2 | 0.1-0.2 | 0.1-0.2 | 0.1-0.2 |
| --- | --- | --- | --- | --- | --- | --- | --- | --- | --- | --- | --- | --- | --- | --- | --- | --- | --- | --- | --- | --- | --- | --- | --- | --- |
|  | Incidence (%) | 15 | 9.9 | 11.5 | 9.7 | 4.3 | 5.5 | 4 | 3.4 | 7.2 | 4.7 | 1.4 | 0.9 | 2.3 | 0.6 | 1.3 | 0.1 | 0.2 | 0.1 | 0.1 | 0.1 | 0.2 | 0.2 | 0.1 |
|  | Prevalence (%) | 45.4 | 43.8 | 41.8 | 44.3 | 38.4 | 18.2 | 19.3 | 23.7 | 20.2 | 21.7 | 5.5 | 3 | 3.8 | 3.4 | 2.5 | 1 | 0.8 | 0.9 | 0.8 | 0.7 | 1 | 1.1 | 0.9 |
|  | BED + (N) | 70 | 32 | 36 | 16 | 19 | 18 | 8 | 9 | 17 | 23 | 11 | 4 | 15 | 4 | 8 | 5 | 14 | 17 | 16 | 16 | 10 | 15 | 18 |
|  | Tested with BED (N) | 381 | 200 | 189 | 80 | 158 | 105 | 57 | 80 | 88 | 162 | 67 | 22 | 50 | 28 | 26 | 43 | 98 | 139 | 103 | 86 | 72 | 108 | 122 |
|  | HIV+ (N) | 557 | 391 | 345 | 218 | 312 | 118 | 81 | 96 | 119 | 179 | 69 | 27 | 52 | 44 | 31 | 49 | 133 | 189 | 173 | 134 | 88 | 170 | 184 |
|  | Participants (N) | 1227 | 893 | 825 | 492 | 813 | 649 | 420 | 405 | 589 | 824 | 1264 | 904 | 1366 | 1313 | 1262 | 4944 | 16644 | 20958 | 21809 | 20467 | 9139 | 15561 | 20763 |
|  | Year | 2004 | 2005 | 2006 | 2007 | 2008 | 2004 | 2005 | 2006 | 2007 | 2008 | 2004 | 2005 | 2006 | 2007 | 2008 | 2004 | 2005 | 2006 | 2007 | 2008 | 2006 | 2007 | 2008 |
|  | Population | Injection drug users | | | | | Discordant couples | | | | | Female sex workers | | | | | Pregnant women | | | | | Pre-marriage couples | | |
