# Supplementary material for: Estimation of HIV-1 incidence among five focal populations in Dehong, Yunnan: a hard hit area along a major drug trafficking route
Source: BMC Public Health. 2010 Apr 7;10:180. doi: 10.1186/1471-2458-10-180 (PMC2858119; doi:10.1186/1471-2458-10-180)
Supplement: Additional file 2 — The proportion of the number of participants with recent HIV-1 infection among HIV-infected participants in Dehong Prefecture, Yunnan Province from 2004 to 2008. Among all HIV-1 positive participants in all groups, the proportion of recent HIV-1 infections among females was higher than that among males. The proportion of recent HIV-1 infections was higher among Han Chinese in 2004 compared to all ethnic minorities, but decreased significantly over time through 2008. Most HIV-1 positive participants were peasants and there was no change over time in the proportion of recent HIV infections. [file 1471-2458-10-180-S2.DOC]

| **The proportion of the number of participants with recent HIV-1 infection among HIV-infected participants in Dehong Prefecture, Yunnan Province from 2004 to 2008** | P^§^ |  |  |  | 0.374 | 0.769 |  | 0.101 | 0.221 | 0.002 |  | 0.962 | 0.037 | 0.08 |  | 0.436 | 0.234 | 0.001 | *: The total number of HIV-infected participants may not equal to the total number of HIV-infected participants because of missing values;  †: HIV(+) No. stands for the total number of HIV-infected participants;  ‡: BED(+) % stands for the proportion of recent HIV-1 infections to the total number of HIV-infected participants;  §: P value indicates the results of trend test. |
| --- | --- | --- | --- | --- | --- | --- | --- | --- | --- | --- | --- | --- | --- | --- | --- | --- | --- | --- | --- |
|  |  | BED(+) | % |  | 7 | 15 |  | 15.25 | 8.33 | 9.09 |  | 10.93 | 10.51 | 7.14 |  | 9.92 | 10 | 15.94 |  |
|  | 2008 | HIV(+) | No. |  | 500 | 340 |  | 223 | 408 | 209 |  | 518 | 257 | 56 |  | 605 | 60 | 69 |  |
|  |  | BED(+) | % |  | 14.14 | 17.82 |  | 15.63 | 7.92 | 11.98 |  | 12.31 | 11.64 | 5.66 |  | 14.81 | 8.24 | 7.61 |  |
|  | 2007 | HIV(+) | No. |  | 198 | 331 |  | 256 | 341 | 167 |  | 325 | 292 | 53 |  | 493 | 85 | 92 |  |
|  |  | BED(+) | % |  | 12.84 | 14.29 |  | 27.14 | 9.18 | 8.07 |  | 10.55 | 15.12 | 10.42 |  | 11.03 | 6.45 | 20.93 |  |
|  | 2006 | HIV(+) | No. |  | 366 | 301 |  | 269 | 392 | 161 |  | 275 | 258 | 144 |  | 517 | 31 | 129 |  |
|  |  | BED(+) | % |  | 12.55 | 13.43 |  | 13.68 | 9.39 | 5.59 |  | 9.19 | 10.09 | 9.09 |  | 9.45 | 6.25 | 18.06 |  |
|  | 2005 | HIV(+) | No. |  | 247 | 201 |  | 212 | 277 | 143 |  | 359 | 218 | 55 |  | 487 | 64 | 72 |  |
|  |  | BED(+) | %^‡^ |  | 12.32 | 15.53 |  | 18.15 | 9.71 | 12.5 |  | 8.35 | 22.75 | 13.41 |  | 8.77 | 16.5 | 30.58 |  |
|  | 2004 | HIV(+) | No.^†^ |  | 568 | 219 |  | 292 | 350 | 152 |  | 479 | 233 | 82 |  | 570 | 103 | 121 |  |
|  |  |  |  |  | Male | Female |  | <25 | 26- | 36- |  | Dai/Jinpo | Han | Other |  | Farmer | Unemployed | Others |  |
|  | Factors^*^ | | | Gender |  |  | Age |  |  |  | Ethnicity |  |  |  | Occupation |  |  |  |  |
